# Concurrent application of TMS and near-infrared optical imaging: methodological considerations and potential artifacts

**DOI:** 10.3389/fnhum.2013.00592

**Published:** 2013-09-19

**Authors:** Nathan A. Parks

**Affiliations:** Department of Psychological Science, University of ArkansasFayetteville, AR, USA

**Keywords:** TMS, near-infrared optical imaging, EROS, fNIRS, connectivity, cortical dynamics

## Abstract

The simultaneous application of transcranial magnetic stimulation (TMS) with non-invasive neuroimaging provides a powerful method for investigating functional connectivity in the human brain and the causal relationships between areas in distributed brain networks. TMS has been combined with numerous neuroimaging techniques including, electroencephalography (EEG), functional magnetic resonance imaging (fMRI), and positron emission tomography (PET). Recent work has also demonstrated the feasibility and utility of combining TMS with non-invasive near-infrared optical imaging techniques, functional near-infrared spectroscopy (fNIRS) and the event-related optical signal (EROS). Simultaneous TMS and optical imaging affords a number of advantages over other neuroimaging methods but also involves a unique set of methodological challenges and considerations. This paper describes the methodology of concurrently performing optical imaging during the administration of TMS, focusing on experimental design, potential artifacts, and approaches to controlling for these artifacts.

## Introduction

Transcranial magnetic stimulation (TMS) is a brain stimulation technique that uses very strong but very brief magnetic fields to induce electrical currents in the human cerebral cortex (Barker et al., 1985; Hallett, 2007). TMS is a unique and powerful tool for human neuroscience as it is the only method capable of focal and non-invasive stimulation of the healthy human brain. TMS provides a method for assessing the causal role of brain regions in cognitive function with considerable spatial and temporal resolution (Pascual-Leone et al., 2000; Walsh and Cowey, 2000). Since the introduction of TMS in the 1980s (Barker et al., 1985), the technique was swiftly adopted by cognitive neuroscientists and has been used widely to study perceptual, motor, and cognitive processes (Pascual-Leone et al., 2000; Walsh and Cowey, 2000; Hallett, 2007).

Much recent methodological development has focused on combining TMS with other neuroimaging techniques (for reviews see Bestmann et al., 2008; Driver et al., 2009; Siebner et al., 2009; Ilmoniemi and Kičić, 2010; Ziemann, 2011). Combined TMS and neuroimaging approaches offer considerable promise in understanding human brain function as a number of novel research questions can be addressed that were previously unapproachable with TMS or neuroimaging alone. First, TMS-neuroimaging provides a method of causally assessing functional connectivity in cortical networks. Measures of functional connectivity in the human brain have traditionally relied on temporal correlations of activity between brain regions (Biswal et al., 1995; Greicius et al., 2003). Such correlational measures of connectivity are unable to provide causal information, rendering it difficult to determine if coactive regions are truly functionally connected or if they merely coincidentally active at similar points in time. Because activations induced in the cerebral cortex propagate trans-synaptically, the concurrent application of TMS and neuroimaging allows functional connectivity between brain regions to be examined in a causal manner (for review see Bestmann et al., 2008; Driver et al., 2009). This is accomplished by using TMS to activate a targeted cortical region while neuroimaging is employed to measure the consequent inter-regional activations. Second, concurrent TMS and neuroimaging provide direct measures of cortical excitability within a targeted brain region. Traditionally, motor-evoked potentials (MEPs), motor thresholds, and phosphene thresholds have been used to index cortical excitability. Combined TMS and neuroimaging approaches can directly measure levels of cortical excitability by evoking a response with TMS while using neuroimaging to measure the magnitude of the resultant activity. Third, TMS-neuroimaging can be applied to better understand mechanisms of neuroplasticity within the human cerebral cortex. Administration of trains of repetitive TMS (rTMS) or patterned rTMS have been shown to modulate states of cortical neuroplasticity (Pascual-Leone et al., 1994; Chen et al., 1997; Huang et al., 2005). Neuroimaging of TMS-induced plasticity provides important insight into neuroplasticity in human cortex. Last, concurrent TMS and neuroimaging provides invaluable information regarding the action of TMS in the human brain, information that previously inferred primarily from MEPs, phosphenes, and animal studies.

To date, TMS has been combined successfully with a number of neuroimaging methodologies including electroencephalography (EEG; Ilmoniemi et al., 1997; Bonato et al., 2006), functional magnetic resonance imaging (fMRI; Bohning et al., 1998, 1999; Baudewig et al., 2001; Bestmann et al., 2004), positron emission tomography (PET; Fox et al., 1997, 2006; Paus et al., 1997), functional near-infrared spectroscopy (fNIRS; Hada et al., 2006; Mochizuki et al., 2006; Kozel et al., 2009), and the event-related optical signal (EROS; Parks et al., 2012). With the fervent development of these combined approaches, it is important to document technical considerations, potential pitfalls, artifacts, and confounds introduced by combining TMS with each of these neuroimaging methodologies. Little information is presently available in the literature concerning the concurrent application of TMS and near-infrared optical imaging methods of fNIRS and EROS. This paper bridges this information gap, giving a brief overview of optical imaging methodologies and their concurrent use with TMS. Although the neurophysiological signals that constitute fNIRS and EROS measurements are quite disparate, the methodological challenges and potential sources of TMS-related artifact are common to the two techniques. Associated design considerations, potential artifacts, and approaches to control for artifacts in TMS-fNIRS and TMS-EROS studies are discussed.

## Near-infrared optical imaging

Non-invasive near-infrared optical imaging refers to a category of neuroimaging techniques that measure activity from the human cerebral cortex through changes in the absorption or scattering of near-infrared light that occur as a consequence of events associated with neural activity. Light in the near-infrared range (650–950 nm) largely escapes absorption by water and is primarily scattered in biological tissue. This scattering permits near-infrared wavelengths to penetrate relatively deep into living tissue. When emitted into the human head, near-infrared light will scatter deeply enough to pass through cortical tissue (Villringer et al., 1993) and a proportion of this light will scatter back to the surface of the scalp where it can be measured by a detector. When the distance between emitter and detector (optodes collectively) is known, the path traveled by light is well-defined, giving near-infrared imaging techniques relatively good spatial resolution. The distance separating a near-infrared emitter and detector determines the depth of penetration into the head (Villringer et al., 1993; Gratton et al., 1995; Gratton and Fabiani, 2010). Consequently, non-invasive near-infrared optical imaging is only capable of measuring the most superficial regions of the cerebral cortex as light intensity becomes insufficient across distances of more than 5.5–6.0 cm. Deep cortical regions, subcortical, midbrain, and hindbrain structures are inaccessible to optical imaging. Thus, near-infrared optical imaging approaches can be used only in the study of cortical function in humans. Methods of near-infrared optical imaging place an array (montage) of fiber optic near-infrared emitters and detectors across the scalp. These emitters and detectors are configured so as to measure activity from cortical regions of interest (ROIs) that lie between an emitter and a detector.

Modulations of certain properties of near-infrared light by the cerebral cortex can be used to infer changes in underlying neural activity. Two classes of signals can be measured using near-infrared optical imaging, a slow hemodynamic response which affects the relative absorption of near-infrared wavelengths and a fast optical signal which affects the scattering properties of near-infrared light. These distinct optical signatures of neural activity form the basis of two non-invasive optical imaging techniques—fNIRS and EROS, respectively.

### Functional near-infrared spectroscopy (fNIRS)

fNIRS is a near-infrared optical neuroimaging method that provides a measure of the hemodynamic response, an increase in blood oxygenation of the cerebral vasculature that peaks 6–10 s following neural activity (Ogawa et al., 1990; Miezin et al., 2000). The hemodynamic response can be measured with near-infrared light because hemoglobin is a primary absorber of near-infrared wavelengths in biological tissue. Short and long near-infrared wavelengths are absorbed differentially by oxygenated hemoglobin (oxy-Hb) and deoxygenated hemoglobin (deoxy-Hb), oxy-Hb absorbing more at longer wavelengths and deoxy-Hb absorbing more at shorter wavelengths (Jobsis, 1977). The changes in oxy-Hb and deoxy-Hb concentrations associated with the hemodynamic response are measureable as modulations in the intensity of light at different near-infrared wavelengths. By obtaining a sparse near-infrared absorption spectrum using two or more wavelengths, it is possible to measure relative concentrations of oxy-Hb and deoxy-Hb within the cerebral vasculature, yielding an index of neuronal activity via the hemodynamic response (Villringer et al., 1993). The spatial resolution of fNIRS (between 1 and 3 cm) is not as good as that of fMRI, the most comparable measure of the hemodynamic response. However, fNIRS has the advantage of acquiring separable measures of oxy-Hb and deoxy-Hb, which are measured only as a composite in the blood-oxygenation level-dependent (BOLD) response. The temporal resolution of fNIRS is limited by the sluggishness of the hemodynamic response, on the order of several seconds. However, the instrumentation of fNIRS generally permits higher temporal sampling of the which allows the hemodynamic response function to be measured with great precision.

### Event-related optical signal (EROS)

In addition to the slow modulations of near-infrared absorption that occur with changes in blood oxygenation, certain optical properties of neural tissue modulate directly in accordance with neural electrical activity. Numerous *in vivo* and *in vitro* studies have demonstrated that near-infrared light scatters less in active neural tissue as compared to tissue at rest, a phenomenon known as the *fast optical signal* (Frostig et al., 1990; MacVicar and Hochman, 1991; Andrew and MacVicar, 1994; Rector et al., 1997, 2005). These changes in scattering occur simultaneously with neural electrical activity and appear to be the result of neurite swelling caused by the migration of water across ion channels (Foust and Rector, 2007; Lee and Kim, 2010). The fast optical signal can be measured non-invasively from the human brain as a reduction in the intensity of light transmission or as an increase in the time-of-flight (delay) of near-infrared light from a source to a detector (Gratton et al., 1995; Wolf et al., 2002; for review see Wolf et al., 2008; Gratton and Fabiani, 2010). Intensity measures can be used to index the fast optical signal because reduced scattering causes less light to scatter back to the surface thus leading to a transient reduction in measured intensity (Gratton et al., 1995). Delay measures can also index the fast optical signal because reduced scattering causes light, on average, to penetrate deeper into the cortex, travelling a slightly longer path, measurable as an increase in phase delay (Gratton and Fabiani, 2003, 2010). These non-invasive signatures of the fast optical signal form the basis of the EROS neuroimaging technique (also fast optical imaging).

The spatial resolution of EROS is approximately equivalent to that of fNIRS though this resolution can be improved significantly with very high density optode arrangements (Gratton and Fabiani, 2003). The advantage of EROS lies in its measurement of the fast optical signal which gives the technique a temporal resolution on the order of milliseconds. The increased temporal resolution of EROS comes at the cost of signal-to-noise ratio, which is quite low compared to other neuroimaging techniques (Gratton and Fabiani, 2003, 2010).

## Concurrent application of TMS and optical imaging: advantages and limitations

The simultaneous application of TMS with near-infrared optical imaging is an appealing marriage of techniques, offering several advantages over other concurrent TMS-neuroimaging approaches. First, near-infrared optical signals are not susceptible to electromagnetic interference from TMS pulses as they are based on measures of light intensity or timing. In contrast, TMS-EEG and TMS-fMRI measure electrical and magnetic fields, respectively, requiring considerable technological innovations to overcome TMS-induced electromagnetic artifact (Siebner et al., 2009). Second, optical imaging methods permit TMS stimulation to any target scalp location and are not constrained by the pragmatics of TMS coil positioning in an MRI scanner. Third, optical methods provide unique measures and insights into cortical functions that are distinct from other neuroimaging technologies. fNIRS provides a measure of both oxy-Hb and deoxy-Hb concentrations, allowing for a more detailed study of the human hemodynamic response evoked by TMS. EROS provides a spatially and temporally resolved measure, allowing the inter-regional dynamics of TMS-evoked cortical activity to be examined. Last, optical imaging equipment has become affordable and commercially available, allowing studies of cortical connectivity, excitability, and dynamics to be readily studied in a laboratory setting.

The feasibility and utility of concurrent TMS-fNIRS has been demonstrated in numerous studies conducted in the motor system (Noguchi et al., 2003; Hada et al., 2006; Mochizuki et al., 2006, 2007; Chiang et al., 2007; Kozel et al., 2009; Groiss et al., 2013) and prefrontal cortex (Kozel et al., 2009; Thomson et al., 2011a,b, 2013). These studies have demonstrated clear modulations in blood oxygenation directly beneath the coil (proximal activations; Noguchi et al., 2003; Hada et al., 2006; Mochizuki et al., 2006; Kozel et al., 2009; Groiss et al., 2013) as well as inter-regionally in cortical areas not directly activated by TMS (distal activations; Mochizuki et al., 2007; Kozel et al., 2009; Groiss et al., 2013). Optical measurement of TMS-evoked hemodynamic responses has been further validated with *in vivo* studies in cats (Allen et al., 2007).

A single study has demonstrated the feasibility of concurrent TMS-EROS and found TMS-evoked activity in the primary motor cortex directly beneath the TMS coil peaking within 16 ms post-pulse and a later activation of contralateral motor cortex occurring within 40 ms of the TMS pulse (Parks et al., 2012). To date, no animal studies have been conducted to examine effects of TMS on the fast optical signal.

There are several limitations associated with combining TMS with non-invasive optical imaging, as compared to other neuroimaging approaches. The primary disadvantage of TMS-optical imaging is the restricted depth of recordings. Both fNIRS and EROS are capable of obtaining functional data from only the most superficial regions of the brain. As such, TMS-induced neural activity can only be examined within the cerebral cortex. TMS-evoked activations within subcortical structures (e.g., basal ganglia) or deep cortical structures (e.g., cingulate) cannot be measured with either fNIRS or EROS. When such ROIs are of interest, optical imaging cannot be used. TMS-fMRI and TMS-PET are currently the only approaches capable of measuring TMS-induced functional activation within such structures.

In addition to the issue of depth, the major limitation of fNIRS is primarily its reliance on the slow hemodynamic response, a problem shared with TMS-fMRI. TMS-fNIRS has relatively good spatial resolution but very poor temporal resolution. This makes TMS-fNIRS well-suited for applications in need of only spatial localization of TMS-evoked responses but prohibits the method from being applied to study neural dynamics. At present, only TMS-EEG and TMS-EROS can yield such temporally precise data in human subjects.

TMS-EROS is also associated with its own set of limitations. The signal-to-noise ratio of EROS is quite low (Gratton et al., 1995; Gratton and Fabiani, 2003, 2010). TMS-EROS requires averaging of between 100 and 300 trials, approximately ten times that of a typical event-related fNIRS study. Given the suggested safety guidelines for the allowable number and frequency of TMS pulses in human subjects, the number of trials required for TMS-EROS averaging can impose significant restrictions on the design of experiments. A further issue of TMS-EROS relates to the supporting literature and prevalence of the technique. Though there is a growing literature applying EROS in the study of human perception and cognition, this literature remains small compared to other neuroimaging techniques. Consequently, there is simply less extant literature from which to draw when designing and interpreting TMS-EROS studies.

Together, TMS-fNIRS and TMS-EROS studies suggest that the concurrent use of TMS and optical imaging can provide useful tools in the study of human cortical connectivity and cerebral dynamics. However, there are several methodological approaches and TMS-related artifacts that should be considered when conducting such studies.

## Design considerations for TMS-fNIRS and TMS-EROS

### Single-pulse vs. repetitive TMS

Single-pulse TMS refers to the intermittent administration of a single magnetic pulse (<1 Hz) to a target region whereas repetitive TMS (≥1 Hz) refers to a train of TMS pulses occurring at a set frequency. In the cognitive neurosciences, single-pulse TMS is often employed to examine the chronometry of a cortical ROI whereas rTMS is used to continuously disrupt a brain area or to induce a lasting change in cortical excitability (Pascual-Leone et al., 2000). The choice to use single-pulse TMS vs. rTMS is driven primarily by the research question being asked. Either single-pulse or rTMS may be an appropriate design choice when using TMS as a method of causally assessing cortico-cortical connectivity but certain considerations should be taken when choosing between single-pulse and rTMS with near-infrared optical imaging.

Single-pulse TMS can be easily integrated into both fNIRS and EROS experiments. The use of single-pulse in a TMS-fNIRS experiment generally requires an event-related design. Such event-related designs often require long inter-trial intervals (20–30 s) in order to allow the hemodynamic response to return to baseline before the start of each trial. These long periods can impose major constraints on the type of questions that may be addressed in a single-pulse TMS-fNIRS study. Single-pulse designs are ideal for TMS-EROS as EROS recordings require precise time-locking and averaging of several hundred trials to increase signal-to-noise ratio. RTMS is most appropriate for use in a block design, measuring changes in cortical activity during trains of rTMS as compared to periods of rest (or sham rTMS). The measurement of the hemodynamic response using fNIRS lends itself well to such designs. Blocked rTMS-fNIRS designs can be successfully implemented to perform studies of cortical connectivity and neuroplasticity during rTMS administration or by performing pre-rTMS and post-rTMS fNIRS measurements. EROS is not easily adapted for use in a typical block design as the fast optical signal is a transient response. However, the temporal resolution of EROS may allow time-locking to the individual pulses within a given rTMS train. Such an approach may be used to study the cumulative effects of TMS pulses or variations in cortical response to individual pulses delivered in a high-frequency rTMS train.

The cumulative effects of rTMS should also be considered when designing a TMS-fNIRS or TMS-EROS. There are well-known modulations of cortical excitability associated with administration of rTMS. For example, low-frequency rTMS (1-Hz) to motor cortex has been shown to induce a lasting reduction in cortical excitability that persists for 15 min or more (Chen et al., 1997). Conversely, high-frequency trains of rTMS can induce a lasting increase in the excitability of motor cortex (Pascual-Leone et al., 1994; Huang et al., 2005). Such modulations of cortical excitability may have undesirable interactions as cortical excitability may vary with the number of TMS pulses administered. Depending on the research question being investigated, such temporal variation may or may not be problematic or confounding. The modulatory effects of rTMS should be carefully considered and weighed against the objectives of the experiment.

### Proximal vs. distal regions of interest

**Figure 1:**
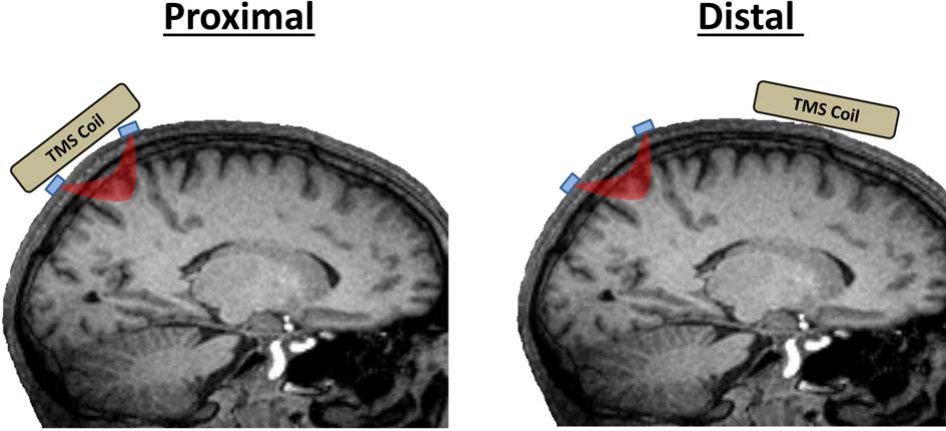
**Proximal vs. distal ROIs in TMS-fNIRS and TMS-EROS experiments.** Proximal ROIs are those directly activated by a TMS coil. Distal ROIs are those activated trans-synaptically by TMS. Optodes are depicted in blue and the path of near-infrared light between an emitter and detector is depicted in red.

TMS-neuroimaging investigations seek to measure TMS-evoked responses from either *proximal* regions of interest (ROIs), those directly beneath the coil, or inter-regional activations that are induced trans-synaptically (*distal* ROIs; Figure 1). Performing near-infrared optical imaging on local vs. distal ROIs is a major determinant of the technical difficulty of an experiment and the TMS-related artifacts that may come into play. Measuring fNIRS or EROS from a proximal ROI directly beneath the coil presents the greatest challenge as optical emitter and detector fibers (optodes collectively) must be designed to accommodate recordings in the presence of a TMS coil. Furthermore, because of coil proximity, proximal ROI recordings are more susceptible to TMS-related artifacts. Further precautions must be taken to control for TMS-related artifacts when recording from proximal ROIs. Distal ROIs may be recorded much more simply in most scenarios without requiring any special modifications to the optical apparatus and with fewer concerns of TMS-related artifacts.

### Optical montage design

**Figure 2:**
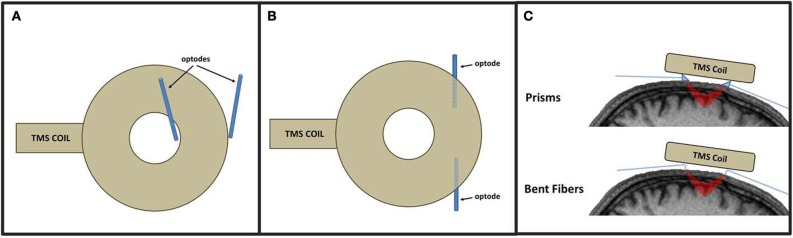
**Approaches to TMS-fNIRS and TMS-EROS montages illustrated with a circular coil.** In a coil-bounding montage **(A)**, optodes are arranges around inner and outer edges of a TMS coil. A low-profile approach **(B)** positions optodes directly beneath the coil by redirecting light with prisms or bending the tips of fiber optics **(C)**.

An optical montage refers to the location and arrangement of emitters and detectors across the scalp to record from the desired cortical ROIs. In a standard TMS-fNIRS or TMS-EROS experiment, optodes are typically secured in place against the scalp with rubber “patches” mounted to the head. For experiments concentrating on distal ROIs, any patch montage can be used so long as the optical mounting apparatus does not limit access of the TMS coil to a targeted scalp location. Performing optical recordings from proximal ROIs presents the greatest technical challenge for TMS-fNIRS and TMS-EROS experiments as the optodes must be mounted against the scalp while still permitting a TMS coil to be positioned close enough to the scalp to activate underlying cortical tissue. The magnetic field generated by a TMS coil falls off rapidly with distance and the introduction of more than a few millimeters of distance can significantly attenuate the efficacy of TMS in underlying cortex (Kozel et al., 2000; McConnell et al., 2001). There are several approaches that may be taken to minimize distance introduced by the placement of an optical patch to measure activity from a proximal ROI. First, a *coil-bounding* optical montage may be incorporated with some TMS coils (Hada et al., 2006). A coil-bounding montage refers to the placement of optodes around the edges or openings of a TMS coil (Figure 2A). Coil-bounding montages are ideal as no additional distance is introduced between the TMS coil and the scalp. The drawback of this approach is that the placement of optodes becomes constrained by the shape of the TMS coil and does not facilitate optimization of a montage for recording a desired ROI. Furthermore, coil-bounding montages are incompatible with many standard TMS coils because distances introduced by the coil are too great to allow light transmission of adequate intensity between emitters and detectors. A second approach for designing a concurrent TMS-optical montage is to reorient optodes so that they may be mounted in a *low-profile* optical patch directly beneath a TMS coil (Figure 2B). Creating a low-profile optical montage can be accomplished either by bending optodes, reorienting their tips at a perpendicular angle (Figure 2C; Noguchi et al., 2003; Mochizuki et al., 2006) or by using prisms to redirect light at a perpendicular angle (Figure 2C; Näsi et al., 2011; Parks et al., 2012). The advantage of the low-profile approach is that a montage may be designed with freedom, arranging optodes in any desired configuration. The disadvantage of such a montage is, of course, the introduction of additional distance between the coil and cortex which may require TMS of greater intensity to sufficiently stimulate the targeted ROI. A final approach for optical imaging of proximal ROIs is to use specialty coils, designed for optical applications. These coils minimize distances between the coil's stimulating “hot spot” and access to the scalp, allowing optodes to be readily positioned in appropriate locations (Groiss et al., 2013). Such specialty coils allow coil-bounding montages to be constructed with greater freedom and across shorter distances than permitted by standard TMS coil designs.

### Safety

To ensure subject safety in TMS-fNIRS and TMS-EROS experiments, optodes and mounting apparatus should be MRI-compatible, free of all ferrous material. Additionally, all components must be thoroughly inspected and tested for materials that may present additionally hazard in the presence of strong alternating magnetic fields, such as metallic materials that may become heated with repeated exposure to TMS pulses. So long as these standards are met, there are no additional safety considerations with TMS-fNIRS or TMS-EROS experiments above those associated with the use of TMS alone (Rossi et al., 2009).

## Potential artifacts in tms-fnirs and TMS-EROS

### Electromagnetic artifacts

**Figure 3:**
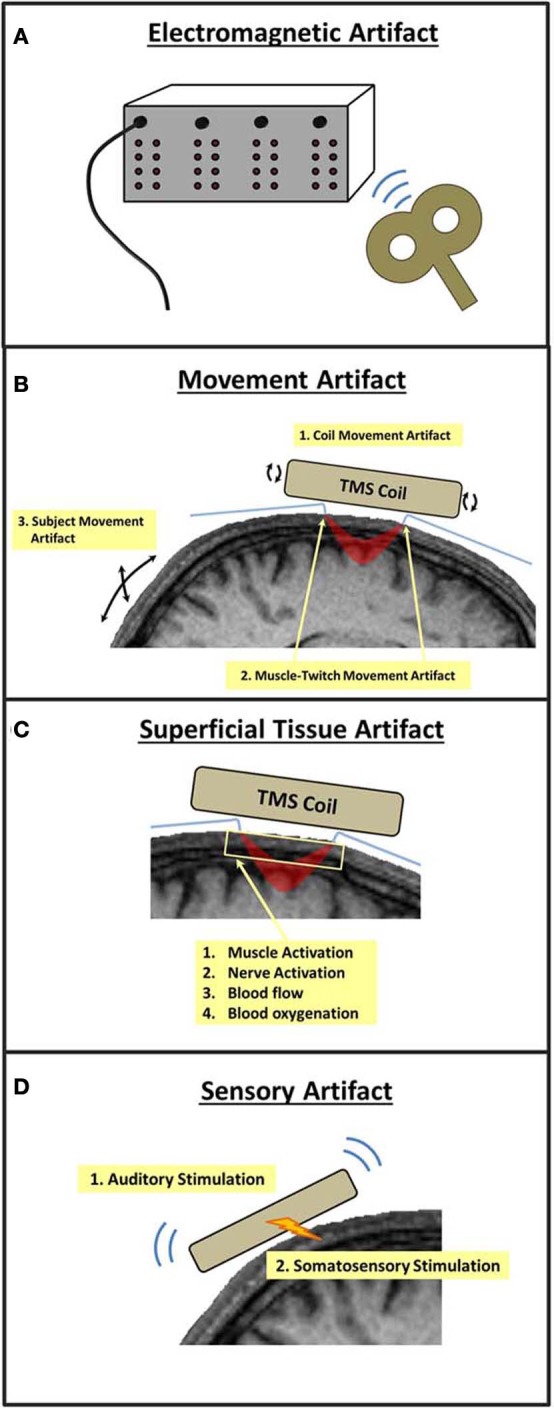
**Potential sources of TMS-related artifacts in TMS-fNIRS and TMS-EROS experiments.** A TMS pulse may **(A)** introduce interference or noise within the optical imaging instrumentation, **(B)** cause movement of the subject and/or optical fibers, **(C)** induce changes in the absorption and scattering properties of superficial tissue of the scalp and skull, or **(D)** induce neural activations through auditory or somatosensory stimulation.

As described previously, one advantage of concurrent TMS and optical imaging is that the measurement of near-infrared light is immune to electromagnetic interference. Though light transmission is not affected by TMS pulses, the instrumentation used to measure near-infrared light may be susceptible to electromagnetic interference, particularly in the sensitive photomultiplier tubes (PMTs) that are used to amplify the detection of near-infrared wavelengths (Figure 3A).

### Movement artifacts

The magnetic discharge of a TMS coil causes mechanical deformation, resulting in small movements and vibrations of the coil. Such vibrations can cause small displacements of optodes or transient changes in the pressure of optodes against the scalp. The presence of either form of mechanical agitation at the optode-scalp interface can introduce brief changes in the measurement of light intensity/scatter that will occur systematically with each TMS pulse (*coil movement artifact;* Figure 3B). Direct stimulation of the scalp musculature directly beneath the TMS coil can introduce transient muscle contractions that can also cause small but significant optode displacements (*muscle twitch artifact*; Figure 3B). Artifacts may also occur as a result of involuntary movements made by the subject due to startle in response to a TMS pulse or through stimulation of cranial nerves and neck muscles. Such *subject movement artifacts* may also lead to large movements of optodes leading to undesirable modulations in intensity/scatter measures (Figure 3B).

### Sensory artifacts

The mechanical deformation of the TMS coil generates a clicking sound, a strong auditory stimulus. The vibrations and scalp stimulation associated with a TMS pulse also cause somatosensory stimulation of the skin and musculature directly below the coil (Figure 3D). As with other TMS-neuroimaging approaches, such sensory artifacts are unavoidable as they are precisely time-locked to a TMS pulse. In many cases, sensory artifact can be ruled out based on the optical ROI recorded. For example, an auditory artifact would not be of major concern when recording fNIRS from primary motor cortex. In other cases, these artifacts must be identified, measured, and ruled out as a source of TMS-evoked cortical activation.

### Superficial tissue artifacts

Near-infrared light must pass through superficial tissue (e.g., scalp) before penetrating into the cortex then pass through this tissue again as it scatters back to the surface where it is detected. As such, non-invasive optical imaging is sensitive to changes in near-infrared absorption and scattering that occur not only in the cerebral cortex, but also in the superficial tissue of the scalp and skull. During passive optical imaging recordings, physiological activity within superficial tissue presents little problem as this activity is random with respect to the stimuli and tasks presented to the subject. However, TMS induces electrical currents not only in the neural tissue of the cerebral cortex but also in superficial tissue, musculature, and nerves of the scalp and skull. Thus, TMS-induced changes in near-infrared absorption and scattering may also occur in superficial tissue, including changes in superficial blood flow and oxygenation (Figure 3C) (Näsi et al., 2011). Such *superficial tissue artifacts*, if present, are not easily disentangled from cortical activations and present the greatest challenge for combined TMS and optical imaging experiments (Näsi et al., 2011).

## Identifying and controlling for artifacts in TMS-fNIRS and TMS-EROS

### Phantom tests

An optical imaging phantom is a block of material with near-infrared scattering properties approximating that of the human head. Phantoms are typically used to test the integrity of optical fibers and instrumentation but can also provide useful information regarding TMS artifacts. In a phantom test for TMS-related artifacts the phantom is configured with the optical montage and optical data is recorded as TMS pulses are emitted. Phantom TMS tests are useful for identifying two classes of artifact: electromagnetic and coil movement artifact.

A simple test for electromagnetic artifact by positioning the coil several centimeters above a phantom and optical montage and discharging a number of TMS pulses. Because optical signals will always remain constant through a phantom and no physical contact is made between the coil and optodes in this configuration, any TMS-locked changes observed in optical signals are most likely attributable to electromagnetic interference within the optical instrument. Electromagnetic artifacts can be dealt with easily by increasing the distance between the optical imaging instrument and TMS coil (e.g., longer fibers) or by electrically shielding the instrument in a grounded Faraday cage enclosure.

A test of coil movement artifacts can also be performed by positioning the TMS coil in physical contact with the phantom and optode mounting apparatus, approximating the conditions of testing on a human subject's head. Assuming no electromagnetic artifacts were found in the aforementioned test, TMS-related effects in near-infrared signals in this case can be attributed to the displacement of optodes by mechanical contraction of the TMS coil. Coil-movement artifact can be minimized by building additional shock absorption into the optode mounting apparatus (e.g., foam padding) or by introducing a small spatial separation between the coil and optodes so that no physical contact is made during experimental testing.

### Sham and control site stimulation

Sham and control site stimulation are standard control conditions for TMS experiments. These conditions should also be incorporated into the experimental design of a TMS-fNIRS or TMS-EROS study to provide an online control for electromagnetic, movement, and sensory artifacts.

Sham stimulation is commonly performed by positioning the TMS coil over the targeted ROI but orienting the coil perpendicular to the scalp so that magnetic field is emitted into the open air rather than stimulating cortical tissue. Sham stimulation can be used to rule out or identify the presence of electromagnetic artifact, coil movement artifact, subject movement artifact, and sensory artifact. Because sham stimulation does not directly stimulate the scalp or cortex, the occurrence of any significant optical effects under these conditions is artifactual and can be attributed to one or more of the aforementioned sources. Phantom tests may be used to further determine the relative contributions of these artifacts.

Control site stimulation involves positioning the TMS coil over a cortical area deemed to be distant and neutral from the targeted region of interest (e.g., vertex). Control site stimulation provides a method of artifact assessment by approximating the stimulating parameters of an active TMS condition without stimulating the ROI of interest. Like sham stimulation, control site stimulation will also capture electromagnetic artifact, subject movement artifact, and sensory artifact. Control site stimulation will more closely match sensory sensations than sham stimulation, providing a better control of sensory artifact. Control site stimulation may also be used to assess the presence of muscle-twitch artifact as it will directly stimulate the scalp musculature. The complication of control site stimulation is that TMS will actively stimulate cortical tissue and thus could potentially induce true cortical activations via trans-synaptic projections to an ROI. Additionally, control site stimulation could induce a greater degree of movement artifact if coil placement results in greater activation of head and neck musculature. A control site must be carefully selected to avoid the induction of subject movement artifact and cortical activations of ROIs.

### Superficial channels

**Figure 4:**
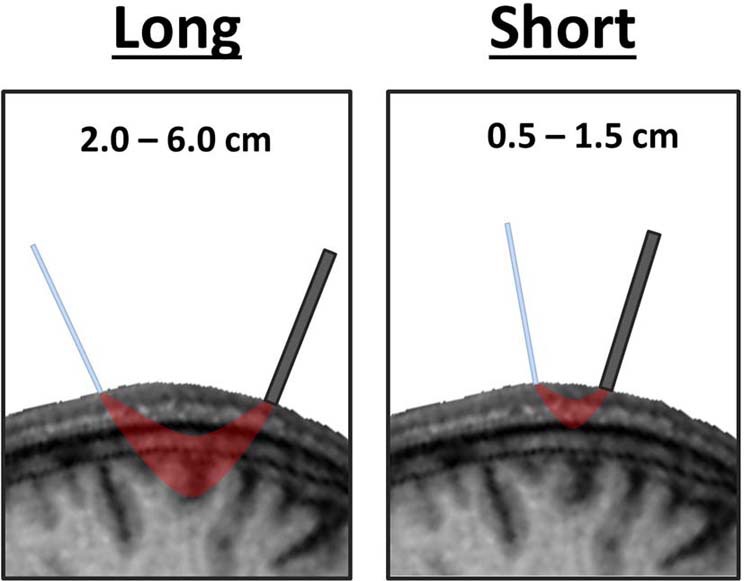
**Varying depth of penetration by near-infrared light between long and short distance channels.** The path travelled by near-infrared light in a channel with long emitter-detector distances passes through cortical tissue whereas the path of light in a short channel travels only superficially through the scalp and skull.

The spatial separation of emitters and detectors determines the depth traveled by near-infrared light. Channels with relatively long source-detector distances penetrate relatively deep into the head, passing through cortical tissue (long channels; ~2.0–5.5 cm; Figure 4). Channels with short source-detector distances penetrate superficially, passing only through the scalp and skull (short channels; <1.5 cm; Figure 4). Though short channels provide no measure of neural activity, they form a powerful method for identifying TMS-related artifacts. This is accomplished by using short channels to yield a measure of near-infrared absorption and scattering that is confined to superficial tissue of the scalp and skull (Firbank et al., 1998; Medvedev et al., 2008; Näsi et al., 2011; Takahashi et al., 2011; Parks et al., 2012; Fabiani et al., 2013). The presence of electromagnetic, movement, and superficial tissue artifacts will be apparent in these channels. The absence (or presence) of effects in short-distance channels as compared to long-distance channels can be used to eliminate (or confirm) the contribution of artifacts as an explanation of observed TMS-evoked fNIRS or EROS activations (Näsi et al., 2011; Parks et al., 2012). Presently, short channels provide the only means of evaluating the contribution of superficial tissue artifacts. Thus, it is critical to incorporate short channels when designing an optical montage for TMS-fNIRS or TMS-EROS experiments so that superficial tissue artifacts may be ruled out when evaluating and interpreting results.

### Coregistration with individual neuroanatomy

Cortical ROIs in near-infrared optical imaging are often localized according to standard scalp locations of the 10–20 system (Jasper, 1958). On average, there is good correspondence between a given 10–20 location and underlying neuroanatomy, allowing near-infrared paths through cortex to be modeled relatively accurately based on these positions alone (Okamoto et al., 2004). However, the spatial accuracy of near-infrared optical imaging can be improved significantly by accounting for individual differences in neuroanatomy by coregistering optode locations to a T1-weighted structural MRI obtained for each subject (Whalen et al., 2008). This coregistration is accomplished by digitizing the three-dimensional locations of optodes at the time of experimentation then using fiducial points and statistical fitting procedures to align optode positions to an anatomical MRI collected for each subject (Whalen et al., 2008; Gratton and Fabiani, 2010). The improved spatial localization gleaned from such coregistration procedures can prove indispensable in disentangling true TMS-evoked cortical activations from sensory artifacts.

### Recording parameters

Electromagnetic and coil movement artifacts will most likely manifest as transient, short-lived modulations in light transmission. In order to capture the occurrence and pattern of transient TMS-induced artifacts, near-infrared optical imaging data should be acquired with the highest sampling rate possible. Minimal filtering should be applied to optical data when identifying potential artifacts, though digital filtering at a later stage of data preprocessing may be an appropriate method to minimize the contribution of certain artifacts.

### Supplementary psychophysiological, psychophysical, and behavioral measures

Simultaneously acquired psychophysiological, perceptual, and behavioral measures can be used to increase confidence in the cortical origins of TMS-evoked fNIRS or EROS activations i.e., MEPs, motor/phosphene thresholds, and response times). A corresponding pattern of effects observed between optical activations and supplementary psychophysiological and behavioral measures is unlikely to be the result of a TMS-related artifact. Appropriate supplementary psychophysiological or behavioral measures should be included whenever allowed by the experimental design and logical given the ROIs being investigated.

### Coil orientation

Systematical manipulation of TMS coil orientation may provide a further method of disentangling superficial tissue artifacts from cortical activation. The orientation of a TMS coil is known to vary the cortical response in systematic ways due to differences in underlying cortical geometry interacting with the direction of induced current flow (Sakai et al., 1997; Di Lazzaro et al., 2004). Although the effects of coil orientation have not yet been investigated with either fNIRS or EROS, coil orientation may predict a relative change in activation magnitude for some ROIs (e.g., motor cortex). Superficial tissue artifacts, however, would not be expected to vary systematically with coil orientation. Thus, optical activations of true cortical origin should modulate in a predictable manner with coil orientation whereas superficial tissue artifacts should not. Demonstrating the optical activation pattern predicted by manipulations of coil orientation could potentially be used to infer the cortical origins of observed signals though, to date, such an approach has not been attempted or validated.

### Activation timing

In some cases, it may be possible to dismiss artifacts based on the timing of optical activations. For example, the temporal resolution of EROS allows cortical activation to be measured within milliseconds of discharging a TMS pulse. TMS-evoked EROS activations were previously described to peak in cortex beneath the TMS coil within 16 ms (Parks et al., 2012). Because this activation preceded the occurrence of an MEP, somatosensory feedback from the MEP could be excluded as a potential explanation of the effect. The temporally delayed effects of the hemodynamic response in fNIRS may also serve as the basis to logically exclude contributions of certain artifacts to optical activations. Electromagnetic and coil movement artifacts, for example, are likely only to induce transient fluctuations in light transmission, which are unlikely to systematically influence measures of oxy-Hb and deoxy-Hb peaking several seconds later.

### Statistical correction of TMS artifacts

Presently, there are no proven methods for filtering or statistical correction of TMS-related artifacts in optical data. However, short-distant channels have been used to understand, measure, and remove superficial signals from fNIRS data (Yamada et al., 2009; Gregg et al., 2010; Takahashi et al., 2011). Independent component analysis has been applied in fNIRS and EROS data to increase signal-to-noise ratio (Kohno et al., 2007; Medvedev et al., 2008). Similar application of such superficial signal regression or blind source separation techniques to concurrent TMS and optical imaging data may provide a future method for the detection and correction of TMS-related artifacts.

## Conclusions

Simultaneous TMS and near-infrared optical imaging provides a flexible and affordable approach to study human cortical dynamics and connectivity. Unique information regarding cortical function, neurovascular coupling, and neuroplasticity can be acquired through measurement of the hemodynamic response (i.e., fNIRS) or fast optical signals (i.e., EROS) concurrently with the administration of TMS. There are a number of technical challenges and TMS-related artifacts associated with TMS-fNIRS and TMS-EROS that must be overcome. In many cases there are straightforward solutions to technical issues and several steps that may be taken to minimize or control for artifactual optical signals.

### Conflict of interest statement

The author declares that the research was conducted in the absence of any commercial or financial relationships that could be construed as a potential conflict of interest.
